# Tax Policy and Total Factor Carbon Emission Efficiency: Evidence from China’s VAT Reform

**DOI:** 10.3390/ijerph19159257

**Published:** 2022-07-28

**Authors:** Da Gao, Xinlin Mo, Ruochan Xiong, Zhiliang Huang

**Affiliations:** 1 School of Literature, Law and Economics, Wuhan University of Science and Technology, Wuhan 430070, China; gaoda@hust.edu.cn; 2 School of Economics, Huazhong University of Science and Technology, Wuhan 430074, China; mxlin@hust.edu.cn (X.M.); m202074251@hust.edu.cn (R.X.)

**Keywords:** Value Added Tax (VAT) reform, total factor carbon emission efficiency, mechanisms

## Abstract

China, the world’s largest carbon emitter, urgently needs to improve its carbon emissions efficiency. This study analyzes the impact of tax policy on total factor carbon emission efficiency (TFCEE). Using the Value Added Tax (VAT) reform in China as an exogenous shock and undesirable-SBM model to measure the total factor carbon emission efficiency of 282 cities in China from 2003 to 2019, our multiple difference-in-difference (DID) estimates show that VAT reform significantly improves the TFCEE in the city level. These potential mechanisms show that VAT reform has promoted upgrading industrial structures, stimulated technological innovation, improved human capital, introduced FDI through four channels, and enhanced the TFCEE. The heterogeneity study found that VAT reform has a higher effect on promoting TFCEE in coastal and large megacities than in inland and small and medium-sized cities. This study provides a theoretical basis for policy instruments to improve energy efficiency and the environment.

## 1. Introduction

China’s economic development is facing the challenges of a slowing growth rate and environmental pollution caused by CO_2_ emissions [1]. As the world’s largest emitter of carbon, China’s carbon dioxide emissions were 9663 million tons in 2018, accounting for 28% of the world’s total carbon dioxide emission, and the problem of carbon dioxide emission in China is becoming more and more serious [2]. The question of how to protect the environment while pursuing high-quality economic growth is an important issue facing China in economic transformation. China’s economic development model can no longer be driven by fixed investment and exports [3,4], but rather by sustainable development that reconciles the economy and the environment. Total factor carbon emission efficiency (TFCEE) is a measure of the efficiency of carbon dioxide emissions from a factor, which is the pollution produced by the factor input, and when the TFCEE is larger, it indicates that less pollution is produced in the manufacturing process. Therefore, improving the TFCEE of enterprises is one of the important indicators to achieve sustainable development goals (SDGs). It is urgent to improve the total factor carbon emission efficiency (TFCEE) to solve the problem of carbon pollution and achieve sustainable economic development.

However, existing scholars have explored how to improve TFCEE in many aspects. For example, studies have found that technological innovation [5,6,7], industrial structure [8,9,10], human capital [11], FDI [3,12], and urban agglomeration [10], etc., are among the more important factors influencing TFCEE. At the same time, some scholars have also investigated the impact of policy instruments on TFCEE, such as the low carbon city policy [12], dual carbon policy [9], and environmental regulation [13]. However, few studies have examined the impact of VAT policy reform on total factor carbon emission efficiency. The most similar study to this paper is Zhou et al. [14], which examines the impact of VAT reform on energy intensity, but this study does not delve into the impact of the carbon emission efficiency of the factors and does not involve a study of the mechanisms of impact. Indeed, VAT reform reduces production costs for enterprises, weakens financing constraints, and increases investment in R&D and innovation, thus possibly promoting the efficient allocation of resources and improving total factor carbon emission efficiency.

Tax policy, an important policy tool for governments, is often used to stimulate investment [15] and total factor productivity [16], promoting economic growth [17]. Few studies have explored the impact of tax incentives that may enhance the TFCEE in urban areas, leading to energy savings and environmental improvements. In 2004, China implemented VAT reform for the three eastern provinces, converting the production tax system into a consumption tax system where companies could deduct the tax on capital goods to stimulate economic development [16]. VAT reform was extended to selected industries in 27 cities in central China in 2007. Then it was extended nationwide in 2009, along with an increased tax on natural resources from 13% to 17%, to achieve environmental protection [18]. As Zhou et al. [14] found, tax incentives affect not only productivity but also the energy efficiency of firms, thus contributing to energy savings. 

Therefore, this paper uses the SBM model to measure China’s urban factor carbon emission efficiency (TFCEE) from 2003 to 2019, using China’s VAT reform as a quasi-natural experiment to investigate the impact of tax policy on TFCEE. Our results show that tax incentives can significantly reduce carbon emission and increase the efficiency of factor carbon emission. Moreover, we further explore the mechanisms of influence and possible heterogeneous effects to explore, systematically and in-depth, the channels through which VAT reform affects TFCEE in cities. The results show that VAT reform has improved the TFCEE by upgrading industrial structures, stimulating technological innovation, improving human capital, and inviting FDI. The heterogeneity study found that VAT reform has a higher effect on promoting total factor carbon emission efficiency in coastal and large megacities than in inland and small and medium-sized cities.

The contribution of our paper is threefold: firstly, we develop total factor carbon emission efficiency (TFCEE) to measure the urban carbon emission efficiency of 282 cities in China from 2003 to 2019 with an undesirable slacks-based model (SBM), which contributes to carbon emission efficiency measurement. Compared to single-factor indicators such as energy consumption intensity such as Zhou et al. [14] and Yu [19], the inclusion of a carbon dioxide indicator in our measure provides a more comprehensive indication of the efficiency of the energy–economy system. 

Secondly, this paper is the first to use China’s VAT reform as a quasi-natural experiment to examine the impact of tax incentives on the total factor carbon emission efficiency at the city level. Previous studies have focused more on the impact of policies on environmental regulation on carbon emission efficiency [9,13], and fewer studies have examined the effect of VAT reform on total factor carbon emission efficiency. In addition, by examining the impact of tax incentives on urban TFCEE with VAT as an exogenous shock, we can overcome the endogeneity problem of the model and help policymakers to assess the real impact of VAT reform better.

Third, we explore the potential mechanisms by which VAT reform affects total factor carbon emission efficiency. There is almost no literature to explore the channels through which policy instruments affect total factor carbon efficiency [14,20]. This paper proposes that tax incentives can promote the total factor carbon efficiency of cities in four ways: promoting industrial upgrading, stimulating technological innovation, improving human capital, and introducing FDI.

The framework for the rest of the paper is as follows. Section 2 presents the background of VAT reform in China, and theoretical analysis of its impact on total factor carbon emission efficiency and its mechanisms. Section 3 introduces the model setting, variables, and data sources. Section 4 presents the empirical results such as baseline results, robustness tests, placebo tests, potential mechanisms, and heterogeneity analysis. Section 5 provides summary conclusions.

## 2. Background and Research Hypothesis 

### 2.1. Background of VAT Reform in China

The VAT reform has been adopted by over 140 developed and developing countries as the most commonly used type of tax [21]. Value Added Tax (VAT) is a tax on the difference between the gross sales and the inputs purchased from other businesses. Purchasing capital goods is usually counted as an input, making VAT a consumption tax. China’s 1994 fundamental tax reform adopted VAT nationwide across all industries, with the tax rate reduced from 17% to 13% to incentivize the extraction of metallic and non-metallic mineral resources and a 0% tax rate on all exports. The VAT adopted in 1994 differs from the traditional consumption-based VAT in one important respect. In calculating a company’s VAT liability, purchases of fixed investments are not deducted from the sale of the final product. In other words, investment goods are subject to VAT twice, first on the final product as its producer and second on the intermediate inputs as its user [7].

In 2004, VAT reform began to be piloted in the three northeastern provinces, with six industries in the manufacturing sector moving from a production tax to a consumption tax, and all investments in transport facilities and fixed assets being deductible from the sale of the final product. In 2007, the VAT reform was extended to 26 cities in central China. It included the mining, power, and energy sectors, with four new cities in Inner Mongolia and 51 counties that were affected by the Wenchuan earthquake being added in 2008. In 2009, the VAT reform was implemented nationwide. The VAT on metallic and non-metallic mining was restored to 17% to increase tax revenues from resource extraction, prevent over-exploitation, and protect the resource environment. (Data from: http://www.gov.cn/jrzg/2008-11/11/content_1146138.htm, accessed on 1 May 2022). We show the VAT reform schedule and the reform cities in Table 1. 

### 2.2. Research Hypothesis 

#### 2.2.1. Basic Hypothesis 

Based on the previous analysis of the background of VAT reform in China, this paper considers that the economic effects of VAT reform may not only affect the behavior of firms in terms of investment [22], productivity [16], and exports [23]. Still, it may also further affect the total factor carbon emission efficiency. Zhou et al. [14] show that VAT reform can significantly promote the energy intensity of firms, but the indicator does not provide a detailed measure of energy efficiency, nor does it further discuss the effects of tax incentives on the efficiency of factor carbon emission. Based on this, this paper proposes that VAT reform may have price, structural, and technology effects that promote the total factor carbon emission efficiency (TFCEE). Firstly, VAT reform increases the price of energy factors by increasing the tax rate of energy factors [24], reducing the market demand for energy factors, and improving the carbon emission efficiency of factors. Secondly, tax incentives can also provide tax rebates for green industries to encourage green technological innovation while imposing high tax rates on high polluting and emitting industries to change the market structure and thus promote total factor carbon emission efficiency. Thirdly, the VAT reform relieves the financing constraints of enterprises [21] and stimulates them to increase their R&D and capital investment in technology and technological innovation. Technological innovation is the main driver of total factor carbon emissions efficiency [25,26], as it increases factor inputs per unit of product and reduces waste gas emissions. Based on the above discussion, we propose the following hypothesis:

**Hypothesis** **1** **(H1).** *The VAT reform has a significant promoting effect on total factor carbon emission efficiency.*

#### 2.2.2. Mechanism Hypothesis 

China’s VAT reform is sector-specific and purposeful. For example, in 2009, the tax on natural resources was increased from 13% to 17% to conserve resources and enhance environmental protection. The change in tax policy affects the demand and supply of mineral resources in the market, thus changing the market price of factors. With the increase in the price of mineral resources, industries would reduce the use of highly polluting elements, pursue the use of green and environmentally friendly factors, change the market structure of factors, and promote the development of industries in the direction of energy conservation and emission reduction. [24]. Upgrading the industrial structural implies a shift in factors of production from low-productivity industries to high-productivity industries [27], thus promoting resource efficiency and improving the total factor carbon emission efficiency [28,29]. Based on the above discussion, we propose the hypothesis:

**Hypothesis** **2** **(H2).** *The VAT reform improves total factor carbon emission efficiency by promoting the upgrading of industrial structures.*

The fact that technological innovation can improve total factor carbon emission efficiency has been proven by many scholars [6,30,31]. China’s tax incentives reduce firms’ production costs by influencing firms’ investment in fixed assets and offsetting firms’ capital purchase taxes [14]. Reducing firms’ production costs can ease firms’ financing constraints, thus allowing more capital to be invested in R&D and innovation, or even the importation of advanced technologies from abroad, which boosts firms’ productivity and technological innovation. Zhang and Wei [26] suggest that the growth in carbon performance is mainly driven by technological innovation. Fang et al. [25] found that technological innovation can improve firms’ labor productivity and reduce factor inputs per unit of product and that firms’ more efficient use of energy factors increases the efficiency of factor carbon emission. Furthermore, Gao et al. [8] also found that green technology innovation can significantly contribute to the green total factor energy efficiency in cities. Similarly, examining the relationship between technological change and energy efficiency, Zhu et al. [32] find that technological innovation can reduce energy consumption and the extent of carbon emission without harming China’s economic growth. Based on the above discussion, we propose the hypothesis:

**Hypothesis** **3** **(H3).** *The VAT reform improves total factor carbon emission efficiency by stimulating technological innovation.*

For firms, the VAT reform can enable enterprises to have sufficient funds so that they can bring in highly skilled personnel and optimize the human capital structure; for the government, VAT reform can stimulate economic development and raise government revenue so that local governments can spend more funds on training highly skilled personnel, improving the productivity of the workforce and optimizing the overall human capital structure of society. With the improvement in the human capital of enterprises and society, the allocation of resources of enterprises and society can be improved, avoiding energy wastage and enhancing the efficiency of factor carbon emission of the whole enterprise and society. As Wang et al. [11] demonstrate, educating human capital can knowledge spillover, industrial upgrading, and environmental regulation to improve total factor carbon emission efficiency. Gao et al. [8] state that the better the human capital, the better the allocation of resources and the savings in production costs for the firm, thus improving the carbon emission efficiency of the factor. Based on the above discussion, we propose the hypothesis:

**Hypothesis** **4** **(H4).** *The VAT reform improves total factor carbon emission efficiency by improving human capital.*

The VAT reform has resulted in cost savings for firms through policies such as lower tax rates and increased subsidies; in other words, the industries involved have gained additional profits and can introduce more foreign investment to produce and operate sales in their domestic markets [7]. The inflow of FDI not only brings advanced production technology and environmental protection concepts from abroad, but the inflow of capital also eases the financing constraints of enterprises and improves their technological progress. At the same time, it will also improve the level of technological innovation in other industries through technological spillover [32]. Furthermore, Hu et al. [33] find that capital-based FDI generates green technology spillovers, which increase green total factor productivity. Similarly, Gao et al. [12] find that FDI can promote green total factor energy efficiency in cities. Based on the above discussion, we propose the hypothesis:

**Hypothesis** **5** **(H5).** *The VAT reform improves total factor carbon emission efficiency by introducing FDI.*

**Figure 1:**
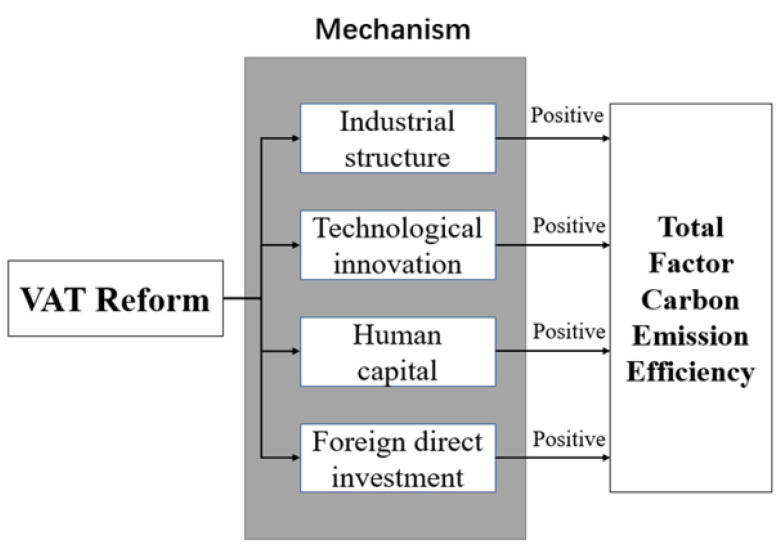
The impact of VAT reform on TFCEE. Source: drawn by the author. As shown above in the mechanism analysis, the impact of VAT reform on TFCEE is mainly through four aspects: industrial structure, technological innovation, FDI, and human capital, and we expect the impact of VAT reform on TFCEE to be positive.

Based on the previous analysis, we graph the impact of VAT reform on TFCEE, as shown in Figure 1.

## 3. Methodology and Data

### 3.1. Model Setting

Considering the possible causal relationship between tax incentives and total factor carbon emission efficiency (TFCEE), we use China’s VAT reform as a policy shock to examine the impact of tax incentives on TFCEE. The VAT reform in China was first piloted in some cities and then fully extended to all cities in the country, and the policy points were 2004, 2007, 2008, and 2009, so we draw on the multi-period approach proposed by Beck et al. [34] to analyze the impact of VAT, and the specific model constructed is shown in Equation (1) below:

(1) $$TFCEE_{it}=\alpha_{1}+\alpha_{2}VAT_{it}+\alpha_{3}{X^{\prime }}_{it}+\gamma_{t}+\theta_{i}+\varepsilon_{it}$$

where $TFCEE_{it}$ represents the total factor carbon emission efficiency of city *i* at time *t.* Dummy variable $VAT_{it}\;$= Treat × Post captures the influence of VAT reform that took place in time *i* on city *c*. When city *i* has performed a VAT reform at time *t*, then $VAT_{it}$ is 1; otherwise it is 0. $\alpha_{2}$ is the estimated coefficient of the effect of VAT reform on $TFCEE_{it}$. $X{’}_{it}$ denotes a range of control variables, including GDP per capita, population density, and sulfur dioxide emission. We also include city-fixed effects $\theta_{i}$ and time-fixed effects $\gamma_{t}$ in the model to control for the city’s invariant and time varying factors. $\varepsilon_{it}$ denotes a random error term.

Based on the discussion of the mechanism analysis in Section 2.2, we would like to examine whether VAT reform affects TFCEE through the human capital, industrial structure, technological innovation, and FDI channels, and the model constructed is shown in Equation (2):

(2) $${Y^{\prime }}_{it}=\beta_{1}+\beta_{2}VAT_{it}+\gamma_{t}+\theta_{i}+\varepsilon_{it}$$

where $Y{’}_{it}$ denotes mechanism variables, which are industrial structural upgrading ($Is_{it}$), technological innovation ($Ti_{it}$), human capital ($Hc_{it}$), and foreign direct investment ($Fdi_{it}$). $\beta_{2}$ indicates the parameter for the effect of VAT on the mechanism variable. $\gamma_{i}$, $\theta_{c}$*,* and $\varepsilon_{it}$ are the same as in Equation (1).

### 3.2. Variables and Data

#### 3.2.1. Variables

##### Dependent Variable

Following the method of Gao et al. [12], we adopt the SBM model to measure the total factor carbon emission efficiency (TFCEE) of 282 prefecture-level cities in China from 2003 to 2019. To be specific, it is assumed that there are N decision-making units (DMU), each of which has M inputs, S1 expected outputs, and S2 unexpected outputs, respectively, which can be expressed in the form of the matrix $X=\left(x_{ij}\right)\in R_{m*n},\;Y^{g}=\left(y_{ij}^{g}\right)\in R_{s1*n},Y^{b}=\left(y_{ij}^{b}\right)\in R_{s2*n}$. The corresponding relaxation vectors of input, expected output, and unexpected output are $S^{-}\in R_{m},\;S^{g}\in R_{s1},\;S^{b}\in R_{s2}$. λ is the weight vector. The calculation formula is as follows:

(3) $$\min p=\frac{1-\left(1/m\right)\sum_{i=1}^{m}s_{i}^{-}/x_{i0}}{1+\frac{1}{s_{1}+s_{2}}\left(\sum_{r=1}^{s1}s_{r}^{g}/y_{r0}^{g}+\sum_{r=1}^{s2}s_{r}^{b}/y_{r0}^{b}\right)}\;s.t.\{\begin{array}{c} x_{0}=X\lambda +s^{-}\\ y_{0}^{g}=Y^{g}\lambda -s^{g}\\ y_{0}^{b}=Y^{b}\lambda -s^{b}\\ \lambda \geq 0,\;s^{-}\geq 0,s^{g}\geq 0,s^{b}\geq 0 \end{array}$$

The measurement of TFCEE mainly includes input, expected output, and undesirable output. The input variables include capital stock, the labor force, and energy consumption. The expected output is economic value, and the undesirable output is CO_2_. Table 2 lists the specific indicators and their explanations.

##### Independent Variable

The core independent variable is the Value Added Tax Reform (VAT), which is a dummy variable. VAT takes the value 1 when city i implements a value added tax reform at time t; otherwise, it takes the value 0. This paper mainly uses four VAT reforms in China in 2004, 2007, 2008, and 2009 as policy shocks.

##### Mechanism Variables

Firstly, the city’s industrial structure reflects mainly agriculture, with manufacturing and services accounting for a significant proportion of total output. The change in industrial structure leads to a change in resource allocation [35], affecting the efficiency of energy use and carbon emissions [36]. We use the share of employment in the tertiary sector to measure changes in industrial structure [8]. Secondly, the level of technological innovation can be measured by the number of patent applications [8]. The number of patent applications reflects the level of innovation, research, and development in a region, which can effectively improve a company’s resource and carbon emission efficiency. Thirdly, we use the number of scientific researchers to measure human capital. Human capital increases a company’s productivity, reduces management costs, and allows for a more optimal resource allocation [11]. Finally, foreign direct investment not only brings capital and advanced technology to firms but also generates technological spillover effects, enhances the technological level of other firms [32] and ultimately enhances the green total factor carbon emission efficiency of the whole region [33].

##### Control Variable

Control variables include population density, GDP per capita, and sulfur dioxide emissions. The selection of control variables was mainly based on [12], where population density was obtained from the ratio of the average number of people in an area to the area. GDP per capita is calculated by dividing the GDP of a region by the number of people in a given period. sulfur dioxide emission represents the pollution level in a city, using the total amount of sulfur dioxide emission in a city in a year.

#### 3.2.2. Data Source and Descriptive Statistics

Our sample is based on data from 2003 to 2019 at the level of 282 prefecture-level cities in China. Data on total factor carbon emission efficiency were obtained from China Urban Statistical Yearbook. Data on energy consumption were obtained from the China Energy Statistical Yearbook. In addition, the mechanism variables and control variables were obtained from the China Urban Statistical Yearbook. Table 3 presents all our variables and includes each variable’s number, maximum, minimum, mean, and standard deviation.

## 4. Empirical Result

### 4.1. The Impact of VAT Reform on TFCEE

To validate the impact of Value Added Tax (VAT) reform on the total factor carbon emission efficiency (TFCEE), we estimate model (1) using a multi-period DID approach. Moreover, to enhance the robustness of the estimation results, we estimated the model with and without control variables separately. The estimated results are shown in Table 4. Column (1) indicates that no control variables were added to the model, and Column (2) indicates that control variables were added to the model, and the estimated parameters of the independent variable VAT are both positive at the 1% level of 0.00459 and 0.0309, respectively. These results suggest that VAT reform significantly contributes to total factor carbon emission efficiency in Chinese cities. The results remain consistent with the inclusion of city control variables and time control variables. Thus, we verify that hypothesis 1 proposed that tax incentives can promote total factor carbon emission efficiency in cities.

The estimated parameters of the control variable GDP per capita are significantly positive at the 1% level, indicating that an increase in GDP per capita can boost the TFCEE of a city. Cities with higher GDP per capita have higher technological levels and productivity, but their environmental awareness will also be more regulated, thus improving the TFCEE [25]. The estimated parameter for SO_2_ is significantly negative, indicating that SO_2_ emissions weaken the city’s TFCEE. More sulfur dioxide emissions indicate that a city is more environmentally polluted and thus less efficient in terms of carbon emission efficiency.

### 4.2. Parallel Trend and Placebo Test

#### 4.2.1. Parallel Trend Test

To ensure the validity of the underlying results, we use a dynamic DID approach to estimate whether a parallel trend existed in the city before the VAT reform. We include a series of year dummy variables to estimate the dynamic impact of VAT reform on urban TFCEE, and the estimated model is shown in Equation (4):

(4) $$TFCEE_{ct}=\alpha_{1}+\alpha_{k}\sum D_{c}^{k}+\alpha_{3}{X^{\prime }}_{ct}+\gamma_{t}+\theta_{c}+\varepsilon_{ct}$$

where $D_{c}^{k}$ is the year dummy variable that equals one when the VAT reform is k years away from city c. Suppose $t_{0}$ is the year in which the city c implemented the VAT reform, then $D_{c}^{k}$ takes a value of 1 when $t-t_{0}=k\;$(*k* = −3, −2, −1, 0, 1, 2, 3) and 0 otherwise. The above model is designed to examine whether there is a parallel trend across all Chinese cities and verify that our main results are valid and unbiased.

**Figure 2:**
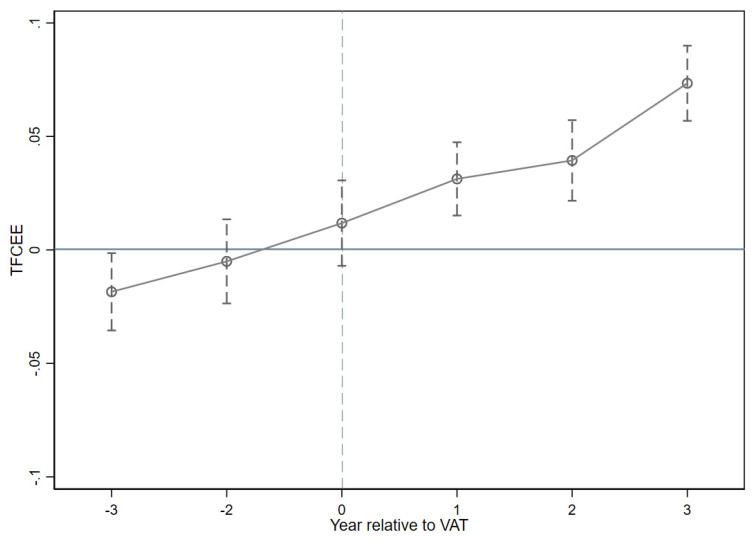
Parallel trend test and the dynamic effect analysis of VAT reform. Notes: The horizontal coordinates indicate the year relative to the reform. Specifically, 0 indicates the year in which the VAT reform took place, and 1 indicates the first year of the VAT reform. The vertical coordinate indicates the size of the dummy variable, with the dashed line depicting the 95% confidence interval. The model also incorporates city and time-fixed effects.

Figure 2 shows the dynamic impact of VAT reform on urban TFCEE with a confidence interval of 95%. We find that the estimated coefficients of our dummy variables are not significantly different from zero in the years before the VAT reform and that there is no significant trend in cities’ TFCEEs for these years, suggesting that the treated and control groups have a parallel trend before the VAT reform. However, following the implementation of the VAT reform, we see a sharp increase in the estimated coefficients of the dummy variables. In Figure 2, the dummy variables $D_{c}^{1}$–$D_{c}^{4}$ are all significantly positive at the 5% level. In other words, city TFCEE increased significantly after implementing the VAT reform. Thus, the results show a parallel trend between the experimental and control groups before the VAT reform, and a significant change in city TFCEE after the VAT reform. This finding confirms the validity and robustness of the impact of the VAT reform on urban TFCEE.

#### 4.2.2. Placebo Test

The results in the base regression find a significant positive effect of VAT reform on urban TFCEE, but there is an artificial selection of pilot cities and many other policy shocks in reality, leading to possible bias in the regression results. In other words, if the pilot cities are non-randomly selected, our results may be caused by some inherent characteristics of the cities rather than by the VAT reform. To address these issues, we use a placebo test for further examination.

**Figure 3:**
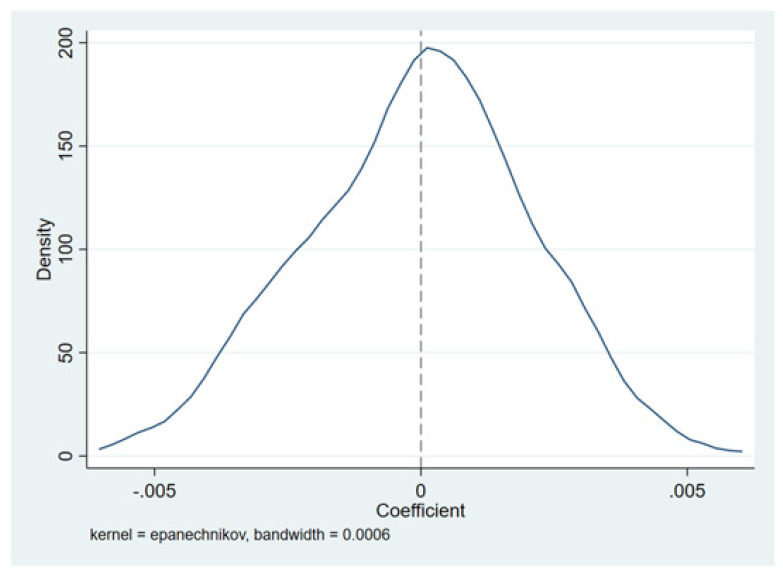
Placebo test for VAT reform randomness.

In the placebo test, we randomize the samples to generate a false-treat group and a false control group. The false VAT is obtained by interacting with the two placebo variables above. We repeat this regression process 200 times but with the false VAT variable, thus evaluating whether the pre-existing factors bias our models. The density distribution of the 200 estimates is shown in Figure 3, where the estimated parameters using the false-VAT variable are close to zero, and the estimated parameters for the previous baseline results are significant for not being 0, indicating that our previous estimates are not due to inherent factors and other shocks.

### 4.3. Robustness Checks

In this section, we perform a series of robustness tests on the baseline results to further enhance the reliability of the conclusions. Firstly, Referring to Cheng et al. [37] and Li and Cheng [5], we first measured the new TFCEE indicators using DDF and EBM and replaced the dependent variables’ indicators before conducting the estimation analysis. Secondly, the VAT reform was carried out nationwide in China in 2009, so we draw on the method of Kong and Xiong [21] to examine only the several VAT reforms before 2009. The robustness results are shown in Table 5.

In Table 5, Columns (1) and (2) indicate the regression results of the TFCEE measured by DDF and EMB, respectively. The estimated parameters in Columns (1) and (2) are 0.0837 and 0.0709, respectively, and both are significant at the 1% level, which indicates that VAT reform has a significant contribution to the city’s TFCEE. In other words, after replacing the dependent variable measure, the estimated results are consistent with the findings of the baseline results. Column (3) indicates the estimated results of VAT reform on urban TFCEE until 2009, and we find that the estimated parameter of VAT is 0.0091 and is significant at the 5% level. This indicates that all phases of VAT reform in China have had a significant contribution to the TFCEE of cities.

In summary, the results of the robustness analysis all suggest that VAT reform has a significant positive impact on urban TFCEE, which is consistent with the previous benchmarking results. This suggests that the conclusions drawn from the benchmark results in this paper are robust and reliable.

### 4.4. Mechanisms Analysis

In Section 4.1, the study finds a significant contribution of VAT reform to urban TFCEE. In this Section, we further explore how VAT reform promotes urban TFCEE. Based on the theoretical assumptions discussed in the previous section, we believe that a potential channel of influence could be that VAT reform may promote industrial structure upgrading, stimulate technological innovation, enhance human capital, and introduce more FDI, thus promoting the city’s TFCEE. Given the large values of the mechanism variable compared to the dependent variable, which may lead to heteroskedasticity in the model, we also treat the mechanism variable logarithmically. The empirical results are shown in Table 6.

Columns (1) and (2) of Table 6 indicate the empirical results for the industry structure variable without and with logarithms, respectively, with estimated parameters of 47,108 and 0.105 for VAT, and both are significant at the 1% level. This indicates that VAT reform significantly promotes industrial structure upgrading in the city, which not only improves productivity but also saves energy and thus promotes TFCEE in the city. The empirical results are consistent with our research hypothesis 2. Columns (3) and (4) show the impact of VAT on technological innovation, with the estimated parameters for VAT both being significantly positive at the 1% level. The empirical results show that the VAT reform has boosted firms’ investment in R&D and technological innovation, thereby significantly increasing their total factor productivity. 

The results in Column (5) and (6) indicate that the estimated parameters for VAT are 0.281 and 0.190 and are significantly positive at the 10% and 1% levels, respectively. This indicates that VAT reform has a boosting effect on human capital regardless of whether the human capital indicator is taken as logarithmic or not. Moreover, when human capital is boosted, it not only boosts the productivity of the city but also saves production costs and improves the use of resources and carbon emission efficiency. Column (7) indicates the effect of VAT on FDI with an estimated VAT parameter of 960,625 and is significant at the 5% level. Column (8) has an estimated parameter of 0.0785, which is insignificant but still positive. This indicates that VAT reform can introduce more foreign investment to the city, which, on the one hand, can alleviate the financing constraint, and on the other hand, foreign investment can bring advanced technology and environmental protection concepts, thus promoting the city’s TFCEE.

Based on the above discussion, the empirical results confirm the mechanism hypothesis in Section 2.2.2, suggesting that VAT reform enhances cities’ TFCEE mainly through four mechanisms: upgrading industrial structure, stimulating technological innovation, promoting human capital, and introducing FDI. The results remain consistent when the mechanism variables are taken logarithmically, indicating that our estimation results are robust and the conclusions are reliable.

### 4.5. Heterogeneity Analysis

In this section, we further analyze whether the heterogeneity of cities leads to a change in the facilitative effect of VAT reform on TFCEE findings. The analysis focuses on heterogeneity in terms of city location and size, which assists us in examining the impact of VAT reform on TFECEE in a more detailed and systematic way.

#### 4.5.1. Location of the City

The effects of VAT reform can be heterogeneous, as cities differ geographically and there are significant differences in transport facilities and environments. Gao et al. [12] found not only significant differences in green total factor productivity between inland and coastal cities, but also that the contribution of FDI to energy efficiency was much higher in coastal cities than inland cities. Therefore, we classify 282 cities in China into coastal and inland cities according to the classification criteria of the China Marine Statistical Yearbook and then examine the impact of VAT reform on urban TFCEE. The empirical results are shown in Table 7 below.

According to Columns (1) and (4) of Table 7, the estimated parameters of VAT for coastal and inland cities are 0.0587 and 0.0434, respectively, and both are significant at the 1% level. This indicates that VAT reform has improved energy efficiency and promoted TFCEE in both inland and coastal cities, but the promotion effect is higher in coastal cities than inland cities. In addition, we used the TFCEE indicators measured by DDF and EBM for the empirical study and the results are presented in Columns (2), (3), (5), and (6), and the findings remain consistent. In other words, VAT reform in coastal and inland cities has catalyzed TFCEE, but the coastal cities have better results.

#### 4.5.2. Scale of the City

The impact of VAT reform on urban TFCEE may vary according to the size of the city. Population agglomeration effects can boost factor productivity by improving energy use efficiency through technological progress [38]. However, Fisher-Vandenet and Ho [39] point out that overpopulated cities can cause urban congestion and increased pollution, which is not conducive to green and sustainable urban development. On this basis, we divide the 282 cities into small and medium-sized cities and large and medium-sized cities according to the State Council’s criteria for classifying the size of cities, with cities with a population of less than 5 million being small and medium-sized cities and those with a population of more than 5 million being mega cities. (Detailed Sources: http://www.gov.cn/zhengce/content/2014-11/20/content_9225.htm, accessed on 24 May 2022). Then, we further examine the heterogeneity of the impact of VAT reform on urban TFCEE across city sizes, with the empirical results shown in Table 8 below.

Columns (1)–(3) of Table 8 show the empirical results of the VAT reform for large and megacities on different measures of TFCEE. We find that the VAT estimates are all significantly positive at the 1% level, suggesting that VAT reform in large and megacities can significantly contribute to the carbon emission efficiency of cities. Columns (4)–(6) of Table 8 show the empirical results of VAT reform in small and medium-sized cities on different measures of TFCEE. We find that the estimates of VAT are also significantly positive at the 1% level. However, comparing the estimated results for the two types of cities, we find that the stimulus effect of VAT reform is much higher in large and megacities than in small and medium-sized cities.

## 5. Discussion

Many previous studies suggest that tax incentives, which are important policy tools for governments, are often used to stimulate investment [15] and total factor productivity [22], thereby promoting economic growth [17]. Few studies have explored the impact of tax incentives that may enhance the total factor carbon emissions efficiency (TFCEE) of cities, leading to energy savings and environmental improvements. This paper uses the SBM model to measure China’s urban factor carbon emission efficiency (TFCEE) from 2003 to 2019, using China’s VAT reform as a quasi-natural experiment to investigate the impact of tax policy on TFCEE. The results of the parallel trend test in Figure 2 found that the total factor carbon emission efficiency increased significantly after the city implemented the VAT reform. In addition, our baseline empirical results in Table 4 show that VAT reform in China significantly promotes urban TFCEE, with estimated parameters of 0.309% and 0.456% for the models with and without control variables, respectively. To ensure that the benchmark results are not affected by measurement errors and other factors, we first measured the new TFCEE indicators using DDF and EBM and replaced the dependent variables’ indicators before conducting the estimation analysis. Secondly, the VAT reform was carried out nationwide in China in 2009, so we draw on the method of Kong and Xiong [21] to examine only the several VAT reforms before 2009. The results of the robustness analysis all suggest that VAT reform has a significant positive impact on urban TFCEE, which is consistent with the previous benchmarking results.

The study by Zhou et al. [14] is the only one in the literature that examines the impact of VAT reform and energy efficiency, but the literature does not further explore the mechanisms of the impact of VAT on energy efficiency or the efficiency of factor carbon emissions. Therefore, we explore how VAT reform promotes urban TFCEE. First, we theoretically discuss potential mechanisms for the presence of VAT on TFCEE in Section 2.2.2. We believe that a potential channel of influence could be that VAT reform may promote industrial structure upgrading, stimulate technological innovation, enhance human capital, and introduce more FDI, thus promoting the city’s TFCEE. The results of the mechanism analysis in Table 6 confirm the theoretical hypothesis, and results remain consistent when the mechanism variables are taken logarithmically, indicating that our estimation results are robust and the conclusions are reliable.

Differences in geographic location and city size may lead to differences in the energy effects of VAT. Gao et al. [12] found not only significant differences in green total factor productivity between inland and coastal cities but also that the contribution of FDI to energy efficiency was much higher in coastal cities than inland cities. Population agglomeration effects can boost factor productivity by improving energy use efficiency through technological progress [38]. However, Fisher-Vandenet and Ho [39] point out that overpopulated cities can cause urban congestion and increased pollution, which is not conducive to green and sustainable urban development. Based on this, we explored whether heterogeneity in geographic location and city size would change the impact of VAT reform on TFCEE. We found VAT reform in coastal and inland cities has catalyzed TFCEE, but the coastal cities have better results, and the stimulus effect of VAT reform is much higher in large and megacities than in small and medium-sized cities.

## 6. Policy Implications

China’s Value Added Tax (VAT) reform, which began in 2004 and finished in 2009, is the reform that has had the greatest influence on China’s tax system. By deducting the cost of capital goods purchased by firms when calculating their value-added tax base, China’s VAT reform affects factor prices, encourages firms to innovate and improve their energy efficiency, and reduces urban carbon emissions.

In view of the above conclusions, this paper puts forward the following policy suggestions. For China to achieve its dual carbon goals (carbon peaking by 2030 and carbon neutral by 2060), the carbon emissions efficiency of its factors must be improved [40]. First, the government should firmly deepen the VAT reform, reduce the production cost of enterprises, and improve the energy efficiency of enterprises and the carbon emission efficiency of factors. Second, enterprises should enhance green technology innovation, vigorously induce foreign direct investment, and introduce high-end technical personnel, so as to enhance the efficiency of enterprise factors carbon emissions, reduce pollution, and improve the ecological environment. Finally, the government should further increase the VAT reform for inland cities and small and medium-sized cities to comprehensively improve the total factor carbon emission efficiency of each city and promote the balanced development of each region.

Our research provides a new perspective on tax policy assessment and contributes to the existing body of research on energy policy. However, there are several limitations to this paper. Firstly, the paper does not examine the costs of VAT reform. Although VAT can reduce cities’ total factor carbon efficiency, government tax revenues may also fall. In addition, some industries with low capacity will be eliminated, which may lead to unemployment. Secondly, this paper only measures the total factor carbon efficiency of cities. In contrast, the total factor carbon emission efficiency at the firm level can give a more accurate picture of the change in factor carbon emissions. Finally, although a series of robustness tests have been carried out in this paper, they all use the fixed effects model. In the future, a random effect model or energy efficiency model can be used for analysis.

## 7. Conclusions

Using total factor carbon emission efficiency (TFCEE) data for 282 cities in China from 2003 to 2019, we apply the multiple DID approach to China’s VAT reform and examine its impact on total factor carbon emission efficiency. Our empirical results show that VAT reform in China significantly promotes urban TFCEE, with estimated parameters of 0.309% and 0.456% for the models with and without control variables, respectively. This finding remains consistent after a series of robustness tests. A plausible mechanism could be that VAT reform promotes TFCEE of cities through four channels: industrial upgrading, stimulating technological innovation, improving human capital, and introducing FDI. We further explored the impact of urban heterogeneity in two ways: city size and geographical location. The results show that VAT reform has a greater effect on promoting TFCEE in larger and coastal cities. These results are similar with those of Zhou et al. [14], which examines the impact of VAT reform on energy intensity in China.

We propose several directions for future research. First, the indicators of total carbon emission efficiency can be more microscopic, such as measuring the total factor carbon emissions efficiency at the industry or enterprise level, which can more accurately measure the change of factor carbon emissions. Secondly, analysis of the costs and benefits of carbon emissions. For example, while VAT reform may improve the problem of factor carbon emissions, it may also result in the closure of heavily polluting enterprises, causing unemployment and other problems [40]. Finally, the energy prices arising from the VAT reform, and thus the change in market and industry structure, also deserve further study.

## Figures and Tables

**Table 1:** Date and city of VAT reform.

July 2004	Liaoning Province, Jilin Province, and Heilongjiang Province.
July 2007	Twenty-six cities located in the middle six provinces. Specifically, the cities are Taiyuan, Datong, Yangquan, and Chang Zhi in Shanxi Province; Hefei, Maan shan, Bengbu, Wuhu, and Huainan in Anhui Province; Nanchang, Ping xiang, Jingdezhen, and Jiu Jiang in Jiangxi Province; Zhengzhou, Luoyang, Jiaozuo, Ping ding shan, and Kaifeng in Henan Province; Wuhan, Huang shi, Xiang fan, and Shi yan in Hubei Province; and Changsha, Zhuzhou, Xiangtan, and Hengyang in Hunan Province
July 2008	Four cities in Inner Mongolia, namely Hulunbuir, Xingan, Tongliao, Chifeng, and Xilingele, and fifty-one counties that suffered from the Wen chuan earthquake. The counties are located in Guangyuan, Mianyang, and Deyang city in Sichuan Province; Longnan city in Gansu province; and Baoji in Shanxi Province
January, 2009	Nation-wide

Notes: The provinces mentioned in the table indicate that all cities in the province have implemented the VAT reform.

**Table 2:** Input and output variables for evaluating the TFCEE.

Input-Output	Variable	Measurement	Unit
Input	Labor force	The total number of employees of each city	10,000 people
	Capital	The capital stock of each city by using the perpetual inventory method	CNY 10,000
	Energy	Total energy consumption of each city	10,000 tons
Desirable Output	Economic value	Real gross domestic production (GDP) of each city treated with the located provincial GDP deflator	CNY 10,000
Undesirable Output	CO_2_	Total carbon emissions of each city	10,000 tons

Notes: (1) When using the perpetual inventory method to calculate the cumulative capital stock, the depreciation rate is set at 10.96%, and the initial capital stock is obtained by dividing the gross capital formation of the first year by 10.96%; (2) Owing to limited data availability, we use the product of the gross regional product of each city and energy intensity of each city to estimate the energy consumption; (3) By referring to the approach in Chen et al. [30], we use satellite image inversion technique to estimate the carbon dioxide emissions of each city.

**Table 3:** Descriptive Statistics.

Variable	Symbol	Obs	Mean	Std. Dev.	Min	Max
Total Factor Carbon Emission Efficiency	TFCEE	4794	0.291	0.106	0.106	1
GDP per capita	Gpc	4776	37,659.013	31,754.193	99	467,749
Industrial structural	Is	4787	256,758.56	453,702.770	8300	6,810,780
Human capital	Hc	4787	1.134	4.050	0.010	106.8
Sulfur dioxide	SO_2_	4609	52,348.155	56,987.843	2	683,162
FDI	FDI	3764	3,926,520.5	12,749,891	0	1.511 × 10^8^
Technological innovation	Ti	4786	3318.954	9663.539	1	166,609
Population density	Pd	3662	425.668	323.456	4.7	2661.54

**Table 4:** The baseline results of the impacts of VAT reform on TFCEE.

Variable	(1)	(2)
	TFCEE	TFCEE
VAT	0.0459 ***	0.0309 ***
	(0.00511)	(0.00450)
Gpc		1.31 × 10^−6^ ***
		(7.30 × 10^−8^ )
Pd		7.24 × 10^−5^ ***
		(1.40 × 10^−5^ )
SO_2_		−1.79 × 10^−7^ ***
		(4.07 × 10^−8^ )
Constant	0.259 ***	0.212 ***
	(0.00366)	(0.00738)
Year-FE	YES	YES
City-FE	YES	YES
Observations	4794	3631
R-squared	0.688	0.794

Notes: Robust standard errors are in parenthesis. Yes means the variable is added to the model. Year-FE indicates time fixed effects, and City-FE indicates city fixed effects. *** indicates significance at the 1% level.

**Table 5:** Robustness tests: based on different measures of TFCEE and policy time periods.

Variable	(1)	(2)	(3)
	TFCEE-DDF	TFCEE-EBM	TFCEE-SBM (year < 2009)
VAT	0.0837 ***	0.0709 ***	0.0091 **
	(0.0059)	(0.0047)	(0.0043)
Constant	0.598 ***	0.339 ***	0.287 ***
	(0.0042)	(0.0034)	(0.0014)
Controls	YES	YES	YES
Year-FE	YES	YES	YES
City-FE	YES	YES	YES
Observations	4794	4794	1974
R-squared	0.764	0.771	0.921

Notes: Robust standard errors are in parenthesis. Yes means the variable is added to the model. Controls indicate a series of control variables. Year-FE indicates time fixed effects, and City-FE indicates city fixed effects. *** indicates significance at the 1% level, and ** indicates significance at the 5% level.

**Table 6:** Analysis of potential impact mechanisms.

Variable	(1)	(2)	(3)	(4)	(5)	(6)	(7)	(8)
	Is	Ln (Is)	Ti	Ln (Ti)	Hc	Ln (Hc)	FDI	Ln (FDI)
VAT	47,108 ***	0.105 ***	2434 ***	0.569 ***	0.281 *	0.190 ***	960,625 **	0.0785
	(14,645)	(0.0125)	(505.2)	(0.0346)	(0.150)	(0.0250)	(422,847)	(0.0512)
Constant	224,038 ***	11.97 ***	1629 ***	5.960 ***	0.939 ***	−1.014 ***	3.323 × 10^6^ ***	13.00 ***
	(10,481)	(0.0089)	(361.5)	(0.0248)	(0.107)	(0.0179)	(278,453)	(0.0339)
Controls	YES	YES	YES	YES	YES	YES	YES	YES
Year-FE	YES	YES	YES	YES	YES	YES	YES	YES
City-FE	YES	YES	YES	YES	YES	YES	YES	YES
Observations	4787	4787	4786	4786	4787	4787	3760	3743
R-squared	0.861	0.966	0.636	0.956	0.817	0.938	0.872	0.936

Notes: Robust standard errors are in parenthesis. ln denotes taking a logarithmic treatment of the variable. Yes means the variable is added to the model. Controls indicate a series of control variables. Year-FE indicates time fixed effects, City-FE indicates city fixed effects. *** indicates significance at the 1% level, ** indicates significance at the 5% level and * indicates significance at the 10% level.

**Table 7:** Heterogeneity test: Coastal and Inland cities.

Variable	(1)	(2)	(3)	(4)	(5)	(6)
	Coastal City	Coastal City	Coastal City	Inland City	Inland City	Inland City
	SBM	EBM	DDF	SBM	EBM	DDF
VAT	0.0587 ***	0.130 ***	0.0867 ***	0.0434 ***	0.0618 ***	0.0835 ***
	(0.0204)	(0.0150)	(0.0166)	(0.0049)	(0.0049)	(0.0066)
Constant	0.289 ***	0.369 ***	0.644 ***	0.253 ***	0.333 ***	0.589 ***
	(0.0144)	(0.011)	(0.012)	(0.0035)	(0.0035)	(0.0045)
Controls	YES	YES	YES	YES	YES	YES
Year-FE	YES	YES	YES	YES	YES	YES
City-FE	YES	YES	YES	YES	YES	YES
Observations	748	748	748	4046	4046	4046
R-squared	0.646	0.817	0.792	0.703	0.742	0.756

Notes: Robust standard errors are in parenthesis. Yes means the variable is added to the model. Controls indicate a series of control variables. Year-FE indicates time fixed effects, and City-FE indicates city fixed effects. *** displays significance at the 1% level.

**Table 8:** Heterogeneity test: City scale.

Variable	(1)	(2)	(3)	(4)	(5)	(6)
	Large Cities and Megacities	Large Cities and Megacities	Large Cities and Megacities	Small and Medium-Cities	Small and Medium-Cities	Small and Medium-Cities
	SBM	EBM	DDF	SBM	EBM	DDF
VAT	0.0820 ***	0.129 ***	0.0900 ***	0.0449 ***	0.0673 ***	0.0843 ***
	(0.0205)	(0.0187)	(0.0171)	(0.0047)	(0.0046)	(0.0061)
Constant	0.233 ***	0.361 ***	0.583 ***	0.260 ***	0.337 ***	0.599 ***
	(0.0149)	(0.0136)	(0.0125)	(0.0035)	(0.0033)	(0.0044)
Controls	YES	YES	YES	YES	YES	YES
Year-FE	YES	YES	YES	YES	YES	YES
City-FE	YES	YES	YES	YES	YES	YES
Observations	357	357	357	4437	4437	4437
R-squared	0.817	0.879	0.897	0.713	0.769	0.754

Notes: Robust standard errors are in parenthesis. Yes means the variable is added to the model. Controls indicate a series of control variables. Year-FE indicates time fixed effects, and City-FE indicates city fixed effects. *** indicates significance at the 1% level.

## Data Availability

Data available on request due to restrictions, e.g., privacy or ethical.

## References

[1] Yu S., Zheng S., Li X., Li L. (2018). China can peak its energy-related carbon emissions before 2025: Evidence from industry restructuring. Energy Econ..

[2] Le Q., Andrew R.M., Friedlingstein P., Sitch S., Hauck J., Pongratz J., Pickers P.A., Korsbakken J.I., Peters G.P., Canadell J.G. (2018). Global Carbon Budget 2018. Earth Syst. Sci. Data.

[3] Huang J., Cai X., Huang S., Tian S., Lei H. (2019). Technological factors and total factor productivity in China: Evidence based on a panel threshold model. China Econ. Rev..

[4] Li K., Lin B. (2017). Economic growth model, structural transformation, and green productivity in China. Appl. Energy.

[5] Li J., Ma J., Wei W. (2020). Analysis and Evaluation of the Regional Characteristics of Carbon Emission Efficiency for China. Sustainability.

[6] Wang W., Xie H., Jiang T., Zhang D., Xie X. (2016). Measuring the Total-Factor Carbon Emission Performance of Industrial Land Use in China Based on the Global Directional Distance Function and Non-Radial Luenberger Productivity Index. Sustainability.

[7] Zhang Y.-J., Sun Y.-F., Huang J. (2018). Energy efficiency, carbon emission performance, and technology gaps: Evidence from CDM project investment. Energy Policy.

[8] Gao Y., Zhang M., Zheng J. (2021). Accounting and determinants analysis of China’s provincial total factor productivity considering carbon emissions. China Econ. Rev..

[9] Guo X., Wang X., Wu X., Chen X., Li Y. (2022). Carbon Emission Efficiency and Low-Carbon Optimization in Shanxi Province under “Dual Carbon” Background. Energies.

[10] Zhang F., Jin G., Li J., Wang C., Xu N. (2020). Study on Dynamic Total Factor Carbon Emission Efficiency in China’s Urban Agglomerations. Sustainability.

[11] Wang M., Xu M., Ma S. (2021). The effect of the spatial heterogeneity of human capital structure on regional green total factor productivity. Struct. Chang. Econ. Dyn..

[12] Gao D., Li G., Li Y., Gao K. (2022). Does FDI improve green total factor energy efficiency under heterogeneous environmental regulation? Evidence from China. Environ. Sci. Pollut. Res. Int..

[13] Curtis E.M., Lee J.M. (2019). When do environmental regulations backfire? Onsite industrial electricity generation, energy efficiency and policy instruments. J. Environ. Econ. Manag..

[14] Zhou Q., Li T., Gong L. (2022). The effect of tax incentives on energy intensity: Evidence from China’s VAT reform. Energy Econ..

[15] Hassett K.A., Hubbard R.G. (2002). Tax Policy and Bussiness Investment. Handb. Public Econ..

[16] Liu Y., Mao J. (2019). How Do Tax Incentives Affect Investment and Productivity? Firm-Level Evidence from China. Am. Econ. J. Econ. Policy.

[17] Hicks M.J., LaFaive M. (2011). The Influence of Targeted Economic Development Tax Incentives on County Economic Growth: Evidence from Michigan’s MEGA Credits. Econ. Dev. Q..

[18] Zhang Z., Guo J.e., Qian D., Xue Y., Cai L. (2013). Effects and mechanism of influence of China’s resource tax reform: A regional perspective. Energy Econ..

[19] Yu H. (2012). The influential factors of China’s regional energy intensity and its spatial linkages: 1988–2007. Energy Policy.

[20] Adua L., Clark B., York R. (2021). The ineffectiveness of efficiency: The paradoxical effects of state policy on energy consumption in the United States. Energy Res. Soc. Sci..

[21] Kong D., Xiong M. (2020). Unintended consequences of tax incentives on export product quality: Evidence from a natural experiment in China. Rev. Int. Econ..

[22] Liu Q., Lu Y. (2015). Firm investment and exporting: Evidence from China’s value-added tax reform. J. Int. Econ..

[23] Li S. (2018). A structural model of productivity, uncertain demand, and export dynamics. J. Int. Econ..

[24] Xu X., Xu X., Chen Q., Che Y. (2015). The impact on regional “resource curse” by coal resource tax reform in China—A dynamic CGE appraisal. Resour. Policy.

[25] Fang C., Cheng J., Zhu Y., Chen J., Peng X. (2021). Green total factor productivity of extractive industries in China: An explanation from technology heterogeneity. Resour. Policy.

[26] Zhang N., Wei X. (2015). Dynamic total factor carbon emissions performance changes in the Chinese transportation industry. Appl. Energy.

[27] Gao Y., Zhang M. (2019). The measure of technical efficiency of China’s provinces with carbon emission factor and the analysis of the influence of structural variables. Struct. Chang. Econ. Dyn..

[28] Gao D., Li Y., Yang Q. (2020). Can pollution charges reform promote industrial SO_2_ emissions reduction?—Evidence from 189 China’s cities. Energy Environ..

[29] Liang G., Yu D., Ke L. (2021). An Empirical Study on Dynamic Evolution of Industrial Structure and Green Economic Growth—Based on Data from China’s Underdeveloped Areas. Sustainability.

[30] Chen Y., Xu W., Zhou Q., Zhou Z. (2020). Total Factor Energy Efficiency, Carbon Emission Efficiency, and Technology Gap: Evidence from Sub-Industries of Anhui Province in China. Sustainability.

[31] Yao X., Zhou H., Zhang A., Li A. (2015). Regional energy efficiency, carbon emission performance and technology gaps in China: A meta-frontier non-radial directional distance function analysis. Energy Policy.

[32] Zhu J., Niu L., Ruth M., Shi L. (2018). Technological Change and Energy Efficiency in Large Chinese Firms. Ecol. Econ..

[33] Hu J., Wang Z., Huang Q., Zhang X. (2019). Environmental Regulation Intensity, Foreign Direct Investment, and Green Technology Spillover—An Empirical Study. Sustainability.

[34] Beck T., Levine R., Levkov A. (2010). Big Bad Banks? The Winners and Losers from Bank Deregulation in the United States. J. Financ..

[35] Bai Y., Deng X., Jiang S., Zhang Q., Wang Z. (2018). Exploring the relationship between urbanization and urban eco-efficiency: Evidence from prefecture-level cities in China. J. Clean. Prod..

[36] Xiong S., Ma X., Ji J. (2019). The impact of industrial structure efficiency on provincial industrial energy efficiency in China. J. Clean. Prod..

[37] Cheng Z., Li L., Liu J., Zhang H. (2018). Total-factor carbon emission efficiency of China’s provincial industrial sector and its dynamic evolution. Renew. Sustain. Energy Rev..

[38] Jiang H., Jiang P., Wang D., Wu J. (2021). Can smart city construction facilitate green total factor productivity? A quasi-natural experiment based on China’s pilot smart city. Sustain. Cities Soc..

[39] Fisher-Vanden K., Ho M.S. (2007). How do market reforms affect China’s responsiveness to environmental policy?. J. Dev. Econ..

[40] Gao D., Li Y., Li G. (2022). Boosting the green total factor energy efficiency in urban China: Does low-carbon city policy matter?. Environ. Sci. Pollut. Res..

